# Critical yield components for achieving high annual grain yield in ratoon rice

**DOI:** 10.1038/s41598-024-74836-0

**Published:** 2024-10-05

**Authors:** Hui He, Linqiong Song, Weiqin Wang, Huabin Zheng, Qiyuan Tang

**Affiliations:** College of Agronomy, Hunan Agricultural University, Changsha, 410128 China

**Keywords:** Main season, Large-panicle cultivars, Ratoon season, Field trials, Plant breeding

## Abstract

Ratoon rice is considered an eco-friendly and resource-efficient method for rice cultivation, providing innovative strategies to mitigate the global food crisis. To clarify the critical yield components for achieving high annual grain yield in ratoon rice, data from 136 widely cultivated rice cultivars were collected through a six-year field experiment. The study analyzed the correlations between yield components and yields for both the main season and the ratoon season, indicating that main yields vary between 5.9 and 10.9 Mg ha^−1^, exhibiting a highly significant positive correlation with spikelets per panicle; ratoon yields range from 1.8 to 7.1 Mg ha^−1^, showing a highly significant positive correlation with panicles per m^2^, grain filling rate, and 1000-grain weight. Path analysis reveals that, in terms of contributing to ratoon yield, the grain filling rate is the most influential component, followed by panicles per m^2^, and 1000-grain weight. Therefore, by selecting large-panicle cultivars in the main season and enhancing panicles per m^2^, grain filling rate, and grain weight in the ratoon season, high annual yield in ratoon rice can be realized.

**Supplementary Information:**

The online version contains supplementary material available at 10.1038/s41598-024-74836-0.

## Introduction

Rice, as one of the most critical global food crops, plays a vital role in ensuring human food security and nutritional health^1^. However, faced with challenges posed by climate change and the growth of the global population, enhancing rice yield and efficiency has become a prominent focus. In this context, ratoon rice has emerged as a sustainable agricultural practice. It utilizes the axillary buds left after the main season rice harvest to sprout new panicles, thereby yielding additional production^2,3^. This practice not only increases the annual yield per unit area but also optimizes resource utilization and reduces planting costs^4^.

Rice yield is determined by its four yield components: panicles per unit land area, spikelets per panicle, grain filling rate, and grain weight. Previous research has indicated that the average main yield ranged from 8.2 to 10 Mg ha^−1^ ^5^. Key factors influencing the main yield of ratoon rice are a subject of debate among researchers. Some studies suggest that panicles per unit land area is crucial^6^, while others emphasize the importance of spikelets per panicle^7,8^; additionally, some research indicates that spikelets per panicle, along with grain weight, play significant roles in determining the main yield^9^; furthermore, factors such as grain filling rate and grain weight are also considered to be important^10^. For the ratoon season, the average yield is between 2.4 and 3.3 Mg ha^−1^ ^11,12^. Critical components for ratoon yield vary among studies, with some emphasizing panicles per unit land area^9,13–15^; while others highlight the importance of both panicles per unit land area and spikelets per panicle^10,14,17^; additionally, spikelets per panicle, panicles per unit land area, and grain filling rate are identified as impacting ratoon yield^4^.

Previous studies have demonstrated inconsistent insights into the determinants of ratoon rice yield and its yield components. This may be due to the limited duration, different cultivars, complex environments, etc. Hence, our study, using 136 widely cultivated rice cultivars over a six-year field experiment in the same region, aimed to obtain more comprehensive results. Aimed to decipher the relationship between the yield components and the yield in ratoon rice, as well as to assess the influence of these components on yield. These findings are instrumental in developing strategies for breeding and growing high-yielding cultivars of ratoon rice.

## Materials and methods

### Experimental site and cultivars

The experiment was conducted from 2016 to 2021 in the northern region of Hunan Province, China, which has a subtropical monsoon climate with a mean daily temperature during the rice growing season ranging from 21.5 °C to 24.9 °C, and an average annual temperature of 16.1 °C to 16.9 °C and annual rainfall of 1230 to 1700 mm. The experimental field has moderate fertility, with convenient irrigation and drainage. In this study, a total of 136 rice cultivars were used for testing. These cultivars were extensively collected and are all approved varieties, voluntarily provided by seed companies and research institutions, which have granted us permission for their use for experimental purposes. All collected varieties comply with the relevant international, national, and/or institutional guidelines, ensuring the legality and ethical integrity of the research. A detailed list of the cultivars can be found in Table S1.

### Field experimental design and agronomic management practice

The experimental design was a randomized block arrangement with three replications per cultivar, each plot measuring 13.4 m^2^; sowing was done around April, and seedlings were transplanted to the field 30 days after sowing at a planting density of 20 cm × 20 cm. The main season lasted 113 days starting from transplantation. Fertilization during this period included nitrogen (N) at a total of 195 kg ha^−1^, phosphorus pentoxide (P_2_O_5_) at 90 kg ha^−1^, and potassium oxide (K_2_O) at 180 kg ha^–1^. The N was split into 90 kg ha^−1^ as base fertilizer, 70 kg ha^−1^ at the tillering stage, and 35 kg ha^−1^as a later addition. The P was equally divided between the base and tillering stages. The ratoon season lasted 61 days, beginning immediately after the harvest of the main season. During this period, the N was applied at 105 kg ha^−1^and the P at 27 kg ha^−1^. The N was equally divided between sprouting fertilizer (20 days after the main season’s panicle initiation, 105 days post-transplantation) and seedling fertilizer, with all the P applied as seedling fertilizer.

At the maturity of the main season, manual harvesting was done by variety with a stubble height of 30 cm. Within three days after harvesting, shallow water irrigation was combined with the application of seedling fertilizer for the ratoon season. Throughout the growth stage, local high-yield rice cultivation techniques were used for the control of diseases and pests like rice blast and leaf folder.

## Observation indexes and methods

All crops were manually harvested and sun-dried for yield calculation. At maturity, 10 holes are selected from each plot, each plot containing two rice plants, to determine the effective panicle number. The Panicles per m^2^ are calculated based on the effective panicle number and plant spacing. The sampled panicl is separated into branch and grain. The grain is further classified into filled and unfilled grain using an FJ-I air blower seed cleaner, and foreign material is removed. The spikelets per panicle, grain filling rate, and 1000-grain weight are measured and recorded. The grain is then dried and weighed. The dry weight of rice from a 5 m^2^ area is measured, and a 100-gram subsample is taken for sun drying. The subsample is then placed in a 105 °C oven until constant weight is reached, and the weight is recorded. The moisture content is calculated as follows: Moisture content (%) = (weight after sun drying - weight after oven drying) / weight after sun drying × 100. Finally, the yield is converted to a 14% moisture content basis.

### Statistical analysis

The yield and yield components data were calculated, processed, and graphically represented using Excel 2010. Path analysis was conducted using DPS, and correlation analysis was performed using SPSS 26.

## Results

**Fig. 1:**
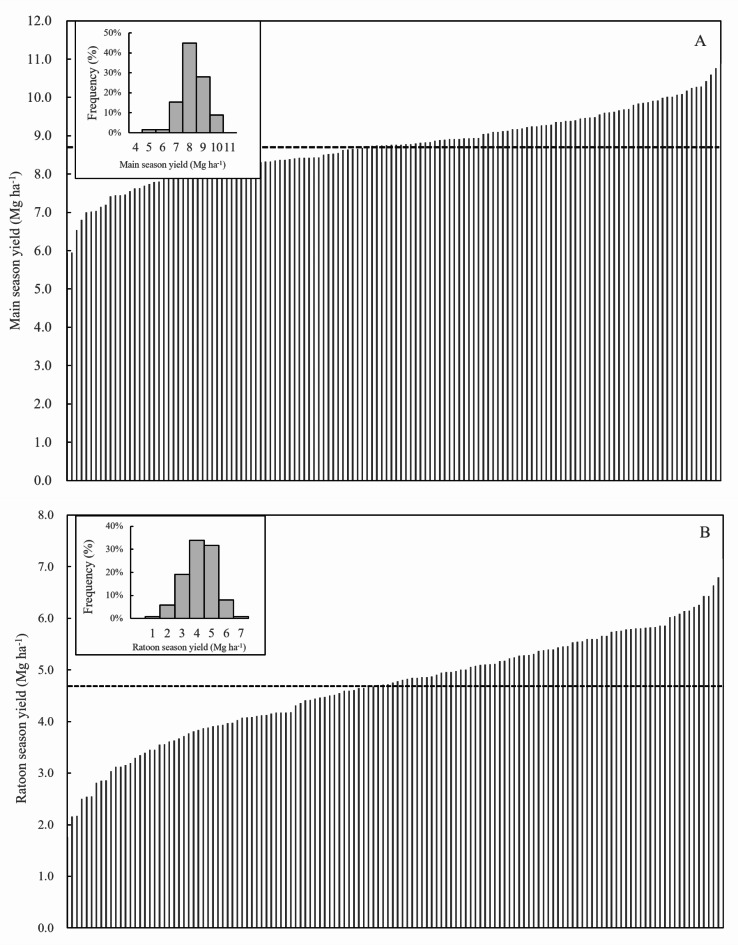
Main yield (**A**) and ratoon yield (**B**). The dashed line horizontally displayed indicates the mean value of the dataset. The smaller figure embedded within illustrates a frequency distribution histogram of the data.

**Fig. 2:**
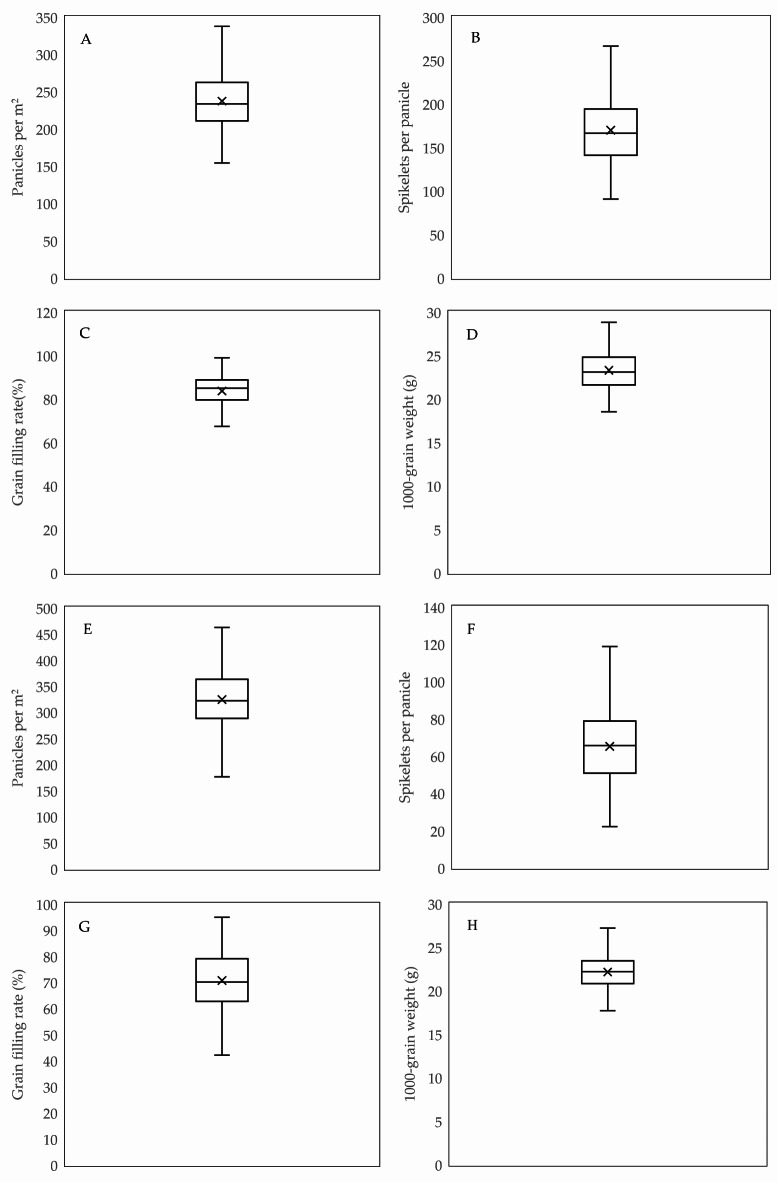
The main yield to panicles per m^2^ (**A**), spikelets per panicle (**B**), grain filling rate (**C**), and 1000-grain weight (**D**); the ratoon yield to panicles per m^2^ (**E**), spikelets per panicle (**F**), grain filling rate (**G**), and 1000-grain weight (**H**). In the box-and-whisker plots, the highest value is represented by the topmost point of the vertical line, the 75th percentile by the upper boundary of the box, the mean by a square inside the box, the 25th percentile by the lower boundary of the box, and the lowest value by the bottommost point of the vertical line.

Following a six-year field experiment, an analysis of data from 136 samples was conducted. The main yields ranged from 5.9 to 10.9 Mg ha^−1^, with an average of 8.7 Mg ha^−1^ (Fig. 1A); panicles per m^2^ ranged from 154 to 350, averaging 237.7 (Fig. 2A); spikelets per panicle varied from 90 to 266, with an average of 169.7 (Fig. 2B); grain filling rate ranged from 67.3% to 98.8%, with an average of 83.6% (Fig. 2C); 1000-grain weight ranged from 15.7 to 28.8 g, with an average of 23.2 g (Fig. 2D). The ratoon yields were 1.8–7.1 Mg ha^−1^, with an average of 4.7 Mg ha^−1^ (Fig. 1B); panicles per m^2^ ranged from 136.0 to 587.3, averaging 333.9 (Fig. 2E); spikelets per panicle varied from 22.0 to 123.0, with an average of 66.0 (Fig. 2F); grain filling rate ranged from 31.6% to 94.9%, averaging 70.3% (Fig. 2G); 1000-grain weight ranged from 13.7 to 27.2 g, with an average of 22.1 g (Fig. 2H).

**Fig. 3:**
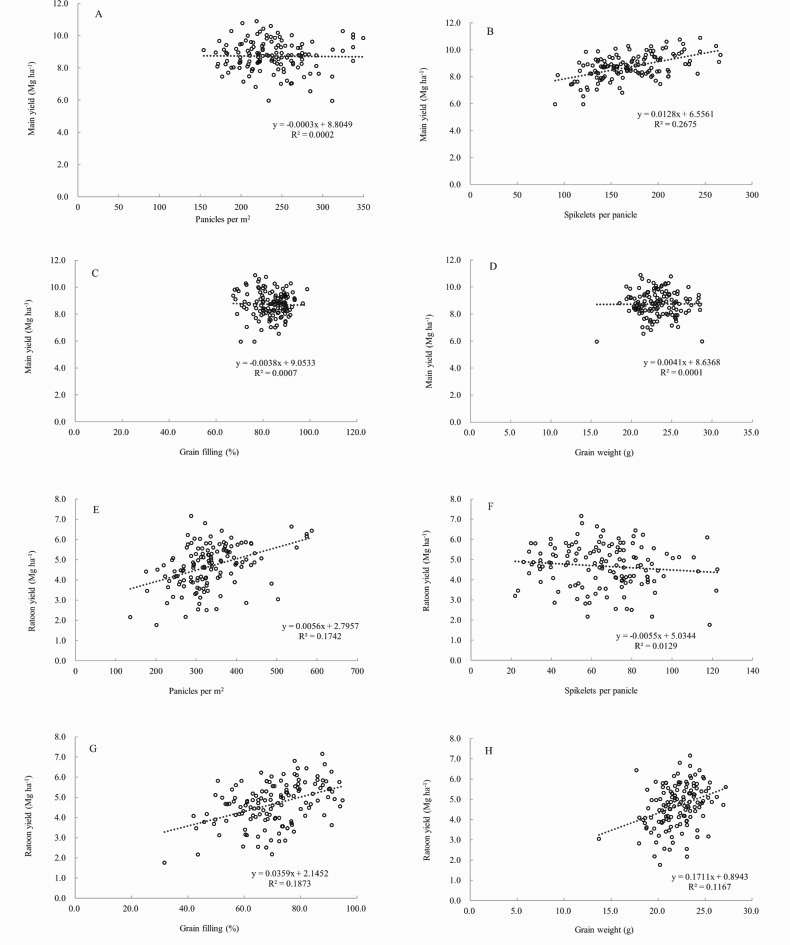
Relationships of the main yield to panicles per m^2^ (**A**), spikelets per panicle (**B**), grain filling rate (**C**), and 1000-grain weight (**D**); relationships of the ratoon yield to panicles per m^2^ (**E**), spikelets per panicle (**F**), grain filling rate (**G**), and 1000-grain weight (**H**).

**Fig. 4:**
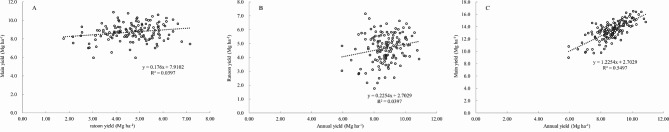
The relationship between main yield and ratoon yield (**A**), relationships of the annual yield to ratoon yield (**B**), and main yield (**C**).

In the main season, only panicles per m^2^ showed a highly significant positive correlation with the main yield (*P* < 0.01) (Fig. 3A-D). Path analysis (Table 1) revealed that spikelets per panicle had the largest direct positive contribution to the main yield (1.075), and even after offsetting by the indirect contributions of the other three yield components, the total contribution remained the highest (0.518). Although panicles per m^2^ had a direct positive contribution to the main yield of 0.620, it was almost offset by the indirect contribution of spikelets per panicle, resulting in a lower total contribution. In the ratoon season, panicles per m^2^, grain filling rate, and 1000-grain weight all showed a highly significant positive correlation with yield (*P* < 0.01) (Fig. 3E-H). Path analysis revealed that the direct contributions of yield components to the ratoon yield were 1000-grain weight (0.405) > panicles per m^2^ (0.374) > grain filling rate (0.278). However, the negative indirect contribution of panicles per m^2^ offset part of the positive direct contribution of 1000-grain weight to the ratoon yield, while the direct contribution of the grain filling rate to ratoon rice, combined with the positive indirect contribution from panicles per m^2^, resulting in total contributions of grain filling rate (0.432) > panicles per m^2^ (0.417) > 1000-grain weight (0.341), aligning with the correlation analysis (Fig. 3E-H).Correlation analysis indicates that there is no significant correlation between the main yield and the ratoon yield, meaning that the ratoon yield is not affected by the main yield. The annual yield of ratoon rice is highly positively correlated with the main yield, while its correlation with the ratoon yield is not significant (Fig. 4A-C).

**Table 1:** Contributions of yield components to the main yield and ratoon yield.

Yield (Mg ha^−1^)	Yield components	Direct contribution	Indirect Contribution via X_i_ (i = 1, 2, 3, 4)				Total contribution
			X_1_	X_2_	X_3_	X_4_	
Main yield	X_1_	0.620		-0.611	0.082	-0.104	-0.013
	X_2_	1.075	-0.352		-0.138	-0.068	0.518
	X_3_	0.311	0.164	-0.476		-0.025	-0.027
	X_4_	0.386	-0.167	-0.188	-0.020		0.010
Ratoon yield	X_1_	0.374		-0.014	0.124	-0.067	0.417
	X_2_	0.034	-0.154		-0.038	0.045	-0.114
	X_3_	0.278	0.167	-0.005		-0.009	0.432
	X_4_	0.405	-0.062	0.004	-0.006		0.341

The factors X_1_, X_2_, X_3_, and X_4_ correspond to panicles per m^2^, spikelets per panicle, grain filling rate, and 1000-grain weight, in that order.

## Discussion

A six-year field experiment involving 136 cultivars of ratoon rice has revealed essential characteristics of their yields. The average yield of the main season stands at 8.7 Mg ha^−1^, aligning with the outcomes of prior studies^12,16^. It indicates that under existing cultivars and cultivation methods, the main yield has reached a stable level. The average yield of the ratoon yield was 4.7 Mg ha^−1^, higher than the previously reported average of 2.4 to 3.3 Mg ha^−1^ ^11,12^. This can be attributed to use the “Four Protections and One Enhancement” cultivation technique developed by the rice cultivation team of Hunan Agricultural University (protection against cold dew wind during the ratoon season’s panicle initiation, high-temperature spikelet initiation and flowering in the main season, lodging, sheath blight, and pests like rice planthoppers, along with enhanced regeneration sprouting ability), which ensured high and stable yield in the ratoon season while optimizing each aspect of rice production and subtly modulating the intrinsic connection between yield components and yield^17^.

This research corroborates that spikelets per panicle were the critical component for the main yield, in agreement with certain studies^9,18^. To improve the main yield, prioritizing the selection and cultivation of large-panicle cultivars is imperative, as these can produce a greater number of grains and thus significantly enhance the main yield^19,20^.

Correlation and path analysis indicated a complex relationship between yield and yield components in the ratoon season, with panicles per m^2^, grain filling rate, and 1000-grain weight showing significant positive correlations with the ratoon yield, differing from previous studies^6,8,14^. Their results showed that the panicles per unit land area^9^, panicles per unit land area and spikelets per panicle^14^, spikelets per panicle, panicles per unit land area, and grain filling rate^8^ were the critical components for the ratoon yield. This variation can be attributed to our study’s use of 136 widely cultivated rice cultivars over a six-year field experiment in the same region, obtaining more comprehensive results. To achieve high yield in the ratoon season, it is crucial to enhance the panicles per unit area, grain filling rate, and grain weight.

Previous research has shown that the main factors influencing the panicles per unit area of the ratoon season include variety type^18^, nutrient management^6,21^, different stubble heights^11,22^, and mechanical compression^23^. To increase the panicles per unit area in the ratoon season, two approaches can be adopted: firstly, selecting varieties with strong regenerative capacity^14^; secondly, implementing effective cultivation measures, including reduced mechanical compression during the main season’s rice harvest^24^; and timely application of sprout-promoting fertilizer^10^.

The heading and grain filling stages are critical periods affecting the grain filling rate and grain weight of the ratoon season. Over 70% of rice yield is derived from dry matter accumulated through leaf photosynthesis during this period^25,26^. Temperature^27^and water management^28^are key factors in the accumulation and transportation of dry matter during those periods, affecting the grain filling rate and grain weight. High temperature reduces the rate of dry matter transport to the panicle, decreasing the accumulation of dry matter in the panicle and thus lowering the grain filling rate^29^. Alternate wetting and drying irrigation can significantly increase the grain filling rate and shorten the grain filling period^30^. This is particularly important for improving the grain weight and uniformity of grain filling in ratoon rice. Moreover, efficient water management can increase grain weigh^31,32^. Therefore, to enhance the grain filling rate and grain weight of the ratoon season, strategies such as water management and avoiding damage from low temperatures during the heading and grain filling stages of ratoon rice can be adopted to promote substance transport.

## Conclusions

Our results were suggested that high grain yield in the main season, ratoon season was related closely with high spikelets per panicle based on six-year field experiment involving 136 rice cultivars. Therefore, an effective way by breeding or selecting large-panicle cultivars for high yield in the main season and pairing them with agronomic measures, higher panicles per unit area, grain filling rate, and grain weight can be achieved in the ratoon season, thereby realizing a high annual yield. Further research is needed to determine tillers dynamic and its effective percentage in the large-panicle rice cultivar under the agronomic measurement include planting density and nitrogen apply rate (recommended: 195 kg ha^−1^ for the main season, 105 kg ha^−1^for the ratoon season), and to define the correlation between panicles per m^2^ and spikelets per panicle.

## Electronic supplementary material

Below is the link to the electronic supplementary material.

Supplementary Material 1

## Data Availability

All obtained data is enclosed with this manuscript [and its supplementary information files].
