# Supplementary material for: Critical yield components for achieving high annual grain yield in ratoon rice
Source: Sci Rep. 2024 Oct 5;14:23190. doi: 10.1038/s41598-024-74836-0 (PMC11455923; doi:10.1038/s41598-024-74836-0)
Supplement: Supplementary file 1 — Supplementary Material 1 [file 41598_2024_74836_MOESM1_ESM.docx]

**Table S1. Details of 136 ratoon rice cultivars in 2016-2021**

| **Year** | **Amount** | **Cultivar** |
| --- | --- | --- |
| **2016** | **23** | Huanghuazhan, Y-liangyou 8188, Y-liangyou 9918, Y-liangyou 7, Y-liangyou 1928, Y-liangyou 828, Longliangyouhuazhan, Jingliangyou Huazhan, C-liangyou 266, C-liangyou 34156, C-liangyou 651, Shenliangyou 5814, Shenliangyou 813, Heyou 328, Taiyou 390, 13ps/0H001, Huyoumingzhan, Weiliangyou 898, Fengliangyouxiang 1, Jinsui 128, Longliangyou 981, Yongyou 4149, Yongyou 2640 |
| **2017** | **32** | Guangliangyou 143, Taoyouxiangzhan, Y-liangyou 551, Pengyou 6377, Liangyou 566, Tianyouhuazhan, Liangyou 389, Wangliangyouhuazhan, Luyou 9803, Dexiang 4103, Mengliangyouhuanglizhan, Liangyou 1876, Xingliangyou 419, Y-liangyou 911, Guangliangyouai 93, Longjingyou 1 Hao, Shenliangyou 475, Meiliangyou 475, Chuangliangyou 33, Jingliangyou 1212, Y-liangyou 143, C-liangyou 343, Huarun 2 Hao, Jiuliangyou 1179, Heyou 50, Quanyousimiao, Liangyou 121, Longliangyou 1988, Yongyou 4949, Wandao 153, Zhunliangyou 608, Huanghuazhan |
| **2018** | **22** | Y-liangyou 9918; Xiangliangyou 143, Longliangyou 1307, Huiliangyou 419, Longliangyou 1319; Heliangyou 1 Hao, C-liangyou 266, Wuyou 61, C-liangyou 0861, Longliangyou 1308, Jingliangyou 1125, Liangyou 867; Wangliangyou 889, Longliangyou 1212; Chuangliangyoufengzhan, Hengfengyouhuazhan, Y-liangyou 2108, Chuanyou 5727, Hanyou 73, Qianfengyou 877, Liangyou 825, Liangyou 1316 |
| **2019** | **21** | Q-liangyou 851, C-liangyou 4418, Meiliangyoujingyinzhan, Y-liangyou 5558, C-liangyou 386, C-liangyou 810, Chuangliangyou 965, Zhenliangyou 749, Jingliangyou 1988, Longliangyou 7810, Yongyou 6760, Huiliangyou 998, Jiuliangyou 1208, Yunliangyou 332, Wangliangyou 911, Hengfengyouyuenongsimiao, Longliangyou 248, Y-liangyou 17, Xingliangyouhuazhan, Yunliangyousimiao, Y-liangyou 9918 |
| **2020** | **19** | C-liangyou 0861, C-liangyou 651, Y-liangyou 551, Y-liangyou 9918, Chuangliangyou 965, Huiliangyou 898, Liangyou 336, Liangyou 389, Longliangyou 1988, Longliangyouhuazhan, Luyou 9803, Shenliangyou 475, Shenliangyou 5814, Shenliangyou 867, Tianyouhuazhan, Wangliangyou 911, Yongyou 4149, Yongyou 4949, Yongyou 4953. |
| **2021** | **19** | Fengliangyou 464; Nanjingxiangzhan; Hengliangyoujinnongsimiao; Wanfengyousizhan; Tianyouhuazhan; Guixiangyoujinnongsimiao; Yexiangyoulisimiao; Shenliangyou 5814; Jingliangyou 1468; Guixiangyouyuzhan; Hengliangyou 19 Xiang; Yexiangyouhaisimiao; Wangliangyou 98 Simiao; Xiangyuezhan; Lvyinzhan; Longliangyouhuazhan; Taiyou 390; Guixiangyou 169; Jiayouchangjing. |
